# Human Hopes

**Published:** 1861-08

**Authors:** 


### HUMAN HOPES.

On the tenth day of December, 1799, " full of health and vigor, Washington looked forward to his long-cherished hope—the enjoyment of a serene old age at Mount Vernon, the home of his heart." On that day he completed a manuscript book of thirty pages, detailing a complete system for the management of his estate for many succeeding years, specifying the cultivation of the several farms, with tables designating the rotation of crops. This manuscript was accompanied by a letter to his manager; this was on Wednesday; on the Saturday following, Washington was dead.

Multitudes are there whose existence is one continued struggle for the means which will enable them to retire to the country and live at their ease for the remainder of life. How few of all that company succeed, need not be expressed here; and of that small number more than half have their hearts so eaten out by the conflicts of life, that no lusciousness is left, no zest for the pure and quiet joys of the country—nothing left but the dry, hard greed of gold, and bitter reflections as to the deep depravity of their fellow men; no sunshine lights up their countenances; no kindly words escape their lips; no generous acts mark out their daily lives; all humanity has died out in them; they have only one joy, and it a semblance—the joy of clutching and hoarding money. Against a life so terrible as this there is a protection—there is a happy deliverance; it consists in wisely enjoying what we may of the present, instead of setting apart a future for it, which we may never, see; all along aiming by word and deed and thought and prayer to secure a resting place in heaven.

